# Investigation of the adolescent female breast transcriptome and the impact of obesity

**DOI:** 10.1186/s13058-020-01279-6

**Published:** 2020-05-11

**Authors:** Adam Burkholder, Dennis Akrobetu, Arun R. Pandiri, Kiki Ton, Sue Kim, Brian I. Labow, Laura C. Nuzzi, Joseph M. Firriolo, Sallie S. Schneider, Suzanne E. Fenton, Natalie D. Shaw

**Affiliations:** 1 Integrative Bioinformatics, National Institute of Environmental Health Sciences (NIEHS), Research Triangle Park, NC USA; 2 Clinical Research Branch, National Institute of Environmental Health Sciences, 111 TW Alexander Drive, MD A2-03, Research Triangle Park, NC 27709 USA; 3 Cellular and Molecular Pathology Branch, Division of National Toxicology Program (DNTP), NIEHS, Research Triangle Park, NC USA; 4 Adolescent Breast Clinic, the Department of Plastic and Oral Surgery, Division of Adolescent/Young Adult Medicine, Boston Children’s Hospital and Harvard Medical School, Boston, MA USA; 5 Biospecimen Resource and Molecular Analysis Facility, Baystate Medical Center, Springfield, MA USA; 6 National Toxicology Program Laboratory, DNTP, NIEHS, Research Triangle Park, NC USA

**Keywords:** Adolescent, Obesity, RNA-seq

## Abstract

**Background:**

Early life environmental exposures affect breast development and breast cancer risk in adulthood. The breast is particularly vulnerable during puberty when mammary epithelial cells proliferate exponentially. In overweight/obese (OB) women, inflammation increases breast aromatase expression and estrogen synthesis and promotes estrogen-receptor (ER)-positive breast cancer. In contrast, recent epidemiological studies suggest that obesity during childhood decreases future breast cancer risk. Studies on environmental exposures and breast cancer risk have thus far been limited to animal models. Here, we present the first interrogation of the human adolescent breast at the molecular level and investigate how obesity affects the immature breast.

**Methods:**

We performed RNA-seq in 62 breast tissue samples from adolescent girls/young women (ADOL; mean age 17.8 years) who underwent reduction mammoplasty. Thirty-one subjects were non-overweight/obese (NOB; mean BMI 23.4 kg/m^2^) and 31 were overweight/obese (OB; BMI 32.1 kg/m^2^). We also compared our data to published mammary transcriptome datasets from women (mean age 39 years) and young adult mice, rats, and macaques.

**Results:**

The ADOL breast transcriptome showed limited (30%) overlap with other species, but 88% overlap with adult women for the 500 most highly expressed genes in each dataset; only 43 genes were shared by all groups. In ADOL, there were 120 differentially expressed genes (DEG) in OB compared with NOB samples (*p*_adj_ < 0.05). Based on these DEG, Ingenuity Pathway Analysis (IPA) identified the cytokines CSF1 and IL-10 and the chemokine receptor CCR2 as among the most highly activated upstream regulators, suggesting increased inflammation in the OB breast. Classical ER targets (e.g., PR, AREG) were not differentially expressed, yet IPA identified the ER and PR and growth factors/receptors (VEGF, HGF, HER3) and kinases (AKT1) involved in hormone-independent ER activation as activated upstream regulators in OB breast tissue.

**Conclusions:**

These studies represent the first investigation of the human breast transcriptome during late puberty/young adulthood and demonstrate that obesity is associated with a transcriptional signature of inflammation which may augment estrogen action in the immature breast microenvironment. We anticipate that these studies will prompt more comprehensive cellular and molecular investigations of obesity and its effect on the breast during this critical developmental window.

## Background

Worldwide, breast cancer is the most common cancer among women and the leading cause of cancer-related deaths in women [1]. Breast cancer rates do not begin to climb until after age 35 years and peak at age 70 years [2], yet there has been a growing recognition that environmental exposures that occur much earlier in life may play important roles in disease pathogenesis [3]. An increased sensitivity to early life exposures relates, in part, to the unique biology of the mammary gland: in contrast to most organs, the mammary gland is immature at birth and undergoes dramatic architectural changes during puberty, pregnancy, and lactation, when full functional differentiation is finally achieved [4].

The exponential proliferative rate of mammary epithelial cells (MECs) that occurs during puberty is believed to make these cells uniquely sensitive to environmental carcinogens during this stage of development [5]. Toxicology studies in rodents and a limited number of epidemiologic studies in populations of girls exposed to chemicals or radiation suggest that adolescence and puberty are in fact critical “windows of susceptibility” to breast cancer [3, 6, 7]. Recent secular changes in the timing and tempo of puberty in girls, including earlier breast development and a longer interval between thelarche and menarche [8–10], are also believed to increase future risk of breast cancer [11, 12]. This is likely due to a prolonged period of unopposed estrogen action (i.e., high levels of estrogen without an accompanying increase in progesterone) on the breast [13] and/or the delayed maturation of sensitive, undifferentiated breast tissue [5].

Changes in the timing of pubertal milestones have occurred in the setting of, and may in part be triggered by, a worldwide pediatric obesity epidemic. This trend is concerning because weight gain during adulthood increases the risk of postmenopausal breast cancer and is associated with higher breast cancer mortality rates in both pre- and postmenopausal women [14]. In obese women, pro-inflammatory factors released by breast adipocytes and stromal cells upregulate aromatase expression, increase local estrogen synthesis, and may thereby promote estrogen-receptor-positive breast cancer development and progression [15]. Somewhat unexpectedly, however, several studies have suggested that during childhood, a higher body mass index (BMI) may actually be associated with a reduced risk of pre-menopausal and postmenopausal breast cancer [16]. The underlying mechanisms driving this unique association between pediatric obesity and future breast cancer risk remain unclear, but a lower breast density among women with a history of childhood obesity has been proposed to be one such mediator [17].

The current studies were designed to investigate the normal mammary transcriptome using RNA-seq and NanoString analyses in adolescent girls and young women to understand how relatively early life factors such as obesity may alter the breast transcriptome at a young age. Datasets such as these will provide insight into how obesity may modulate susceptibility to breast cancer in adulthood.

## Methods

### Breast tissue specimen collection

Sixty-two breast tissue samples were obtained from adolescent girls and young women aged 14–23 years (hereafter termed ADOL) at Baystate Medical Center (Springfield, MA), Boston Children’s Hospital (Boston, MA), or from four participating sites of the NCI-funded Collaborative Human Tissue Network (CHTN) (University of Pennsylvania, Philadelphia, PA; Nationwide Children’s Hospital, Columbus, OH; University of Virginia, Charlottesville, VA; and Vanderbilt University, Nashville, TN) (Additional File 1: Table S1). Samples represented histologically normal tissue collected during reduction mammoplasty in patients with macromastia except for one sample that was obtained as part of bilateral mastectomy for gender reassignment and one sample obtained during removal of a juvenile fibroadenoma. Adolescent girls (< 20 years) with an age-adjusted BMI percentile > 85 and young women with BMI > 27 kg/m^2^ were classified as overweight/obese (OB).

Samples were snap-frozen in liquid nitrogen or placed in dry ice immediately after surgery and were stored at − 80 °C until analysis. Paired paraffin-embedded samples were also available for five subjects.

**Fig. 1:**
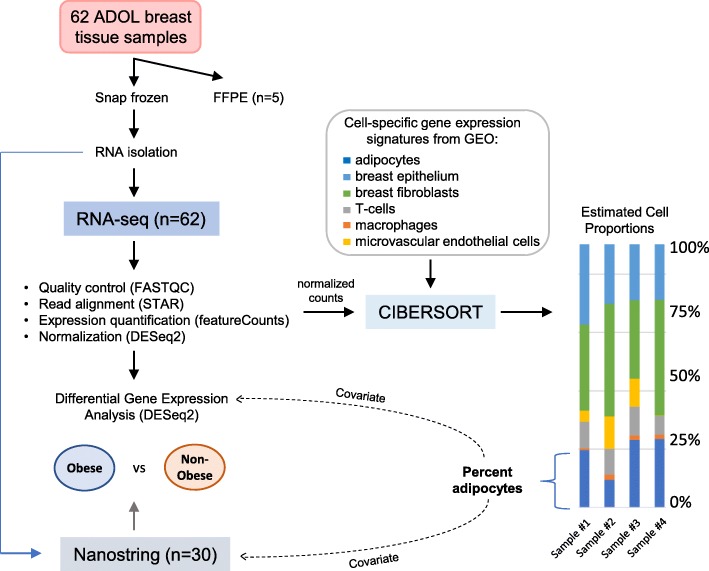
Protocol schema

Tissue collection was approved by the Institutional Review Board at each institution. Informed consent was obtained from all patients (and from a parent or guardian for participants < 18 years old) for future research use of excess tissue not needed for diagnostic purposes. The NIH Office of Human Subjects Research Protections reviewed the protocol components to be completed at NIH (RNA isolation, transcriptome analyses, and immunohistochemical studies in de-identified samples) and concluded that these activities are excluded from IRB Review per 45 CFR 46 and the NIH policy for the use of specimens/data. The complete protocol schema is depicted in Fig. 1.

### Isolation of RNA

ADOL frozen tissue (50 mg) samples were lysed and homogenized in QIAzol lysis reagent (Qiagen Sciences, Inc., Germantown, MD) using a rotor-stator homogenizer. RNA was extracted using the Qiagen miRNAeasy Mini kit (catalog no. 217004) following the manufacturer’s protocol. On-column deoxyribonuclease (DNase) treatment was performed using the Qiagen RNAse-Free DNase Set kit (catalog no. 79254) to purify the RNA samples. RNA concentration and quality were measured on a 4200 TapeStation system (Agilent Technologies, Santa Clara, CA).

### Preparation of cDNA libraries for RNA-seq analysis

ADOL libraries were prepared using the Illumina Tru-Seq RNA kit according to the manufacturer’s instructions. Sequencing was performed for all 62 ADOL samples on the Illumina NovaSeq (NIEHS Epigenomics Core). All sequenced reads were 75 bp and single-end, generating an average read depth of 64 million reads per sample. Sequencing quality was confirmed using FASTQC [18]. Raw reads were aligned to the hg19 reference genome using STAR 2.6.0c [19] and assigned to GENCODE v27 gene models using featureCounts, a component of Subread 1.5.1 [20].

### NanoString nCounter platform

mRNA expression in a subset of ADOL samples (*n* = 30) was measured using the NanoString platform with a custom code set of 69 human endogenous RNA transcripts (Additional File 1: Table S2). The NanoString nCounter uses multiplexed nucleotide probes, labeled with a fluorophore, to generate counts for each gene, as previously described [21]. RNA (50 ng) was prepared as detailed above. RNA expression was quantified on the nCounter Digital AnalyzerTM, and raw and adjusted counts were generated with nSolver (v4.0) TM software. Data were adjusted utilizing six housekeeping genes (*ACTA2*, *ACTB*, *GAPDH*, *RPL19*, *RPLP0*, *TBP*) that were incorporated into the panel. The selected housekeeping genes had minimal variability across all samples and spanned the count range of the dataset. All samples passed nSolver’s initial QA/QC controls. Compiled raw and data adjusted with positive/negative controls and housekeeping genes were exported as .csv files.

### RNA-seq analyses

Counts were imported into DESeq2 1.18.1 [22], run within R 3.4.0, and normalized to account for differences in sequencing depth. Principal component analyses (PCA) were performed using DESeq2 after transformation via the *vst* (variance-stabilizing transformation) function. Recognizing the potential for differences in cellular composition among ADOL samples to bias differential gene expression analyses of non-overweight/obese (NOB) vs. OB samples, normalized read counts from ADOL samples were first exported into CIBERSORT [23]. CIBERSORT uses deconvolution analysis to estimate cell fractions in bulk RNA-seq data. For this analysis, a signature for adipocytes, macrophages, T cells, microvascular endothelial cells, luminal epithelial cells, and mammary fibroblasts was generated based on published RNA-seq studies (Additional File 1: Table S3). For each cell-specific dataset, reported counts per sample were determined by gene symbol (values were summed in cases where multiple identifiers were associated with a single symbol) and normalized jointly using the method applied by DESeq. A CIBERSORT deconvolution analysis was also applied to RNA-seq read counts for breast or for subcutaneous adipose tissue from adult women, aged 20–49 years, acquired from the Genotype-Tissue Expression Portal (GTEx) [24] (Additional File 1: Table S4).

Differential gene expression analysis was completed in DESeq2 to test the effect of ADOL OB vs. NOB body type while considering the CIBERSORT-estimated proportion of adipocytes as a covariate in the design matrix. Differentially expressed genes (DEGs) were accepted based on a false discovery rate (FDR)-adjusted *p* value < 0.05. DEGs were examined for enrichment of canonical pathways and networks as well as for common upstream regulators using Ingenuity Pathway Analysis (v.43605602) [25] and/or the DAVID Bioinformatics Resources Functional Annotation Tool (v6.8) [26].

Heatmap visualizations were based on normalized and transformed counts for ADOL breast samples after adjustment for CIBERSORT-estimated adipocyte proportion using the *removeBatchEffect* from limma (v3.34.9) R package [27]. All heatmaps were generated using Partek Genomics Suite 6.6 (Partek, St. Louis, MO).

### NanoString analyses

NanoString log2 fold changes in ADOL samples were estimated using limma-voom, based on a similar design matrix to that applied in the RNA-seq differential gene expression analysis. Adipocyte proportions, as estimated by CIBERSORT based on RNA-seq data from a matched tissue sample, were included as a covariate for each sample.

### Cross-species comparisons

Microarray expression data for young adult mouse, rat, and macaque mammary tissue were obtained from the Gene Expression Omnibus (GEO) database (Additional File 1: Table S5). The 500 most highly expressed genes were identified in these datasets as well as in the RNA-seq datasets in ADOL samples and adult breast GTEx samples. Intersections among the top 500 gene lists in human, mouse, rat, and macaque mammary tissue were determined using the NCBI Homologene database (downloaded 10/2019). Intersections were accepted on the basis of an identical gene symbol match or identical match with a defined homolog. Euler diagrams depicting intersections were generated using eulerr v5.1.0 [28].

### Estimation of the immune cell fraction in ADOL breast tissue

As previously noted, normalized RNA-seq read counts from ADOL samples were exported into CIBERSORT to estimate cell fractions of macrophages and T cells. Cell fractions were compared between NOB and OB using Fisher’s exact test. Counts were also uploaded into xCell to estimate immune cell counts (34 cell types) and an overall immune score [29] through cell type enrichment analysis. Cell counts or scores were compared between OB and NOB using a Mann-Whitney *U* test.

### Immunohistochemical studies and imaging

The 5 paired ADOL tissue samples were formalin-fixed, paraffin-embedded, and sectioned. Immunohistochemical stains were performed with either a monoclonal mouse anti-human estrogen receptor alpha (ER-α) antibody [6F11] (1:50 dilution, BioRad, Hercules, CA) or a monoclonal mouse anti-human CD68 antibody (1:200 dilution, Dako, Carpenteria, CA). ER-α staining was performed using the ImmPRESS anti-mouse IgG horseradish peroxidase (HRP) polymer (Vector Laboratories, Burlingame, CA), and CD68 staining was performed using a standard avidin-biotin-peroxidase technique. Antigen-antibody complexes were visualized using 3-diaminobenzidine (DAB) chromagen (DakoCytomation, Carpenteria, CA). Slides were scanned at 40× using the Aperio® AT2 Digital Whole Slide Scanner (Leica Biosystems, Buffalo Grove, IL) and visualized using the Aperio® ImageScope v. 12.4.0.7018 (Leica Biosystems).

## Results

### Subjects

For the ADOL dataset, breast tissue samples were collected from 31 NOB subjects (mean ± SD; BMI 23.4 ± 2.0 kg/m^2^) and 31 overweight/OB subjects (BMI 32.1 ± 4.4 kg/m^2^ where OB was defined as a BMI percentile > 85 in girls or BMI > 27 kg/m^2^ in young women) (Additional File 1: Table S1). The two groups were matched for age (NOB 17.7 ± 1.6 vs. OB 17.9 ± 2.7 years; *p* = 0.7), but non-Hispanic Caucasian subjects tended to be over-represented in the NOB group (*p* = 0.07). Two subjects were primiparous, and parity was unknown for one subject; the remainder were nulliparous (Additional File 1: Table S1).

### Breast whole transcriptome profiles during adolescence: young women compared with other mammalian species

**Fig. 2:**
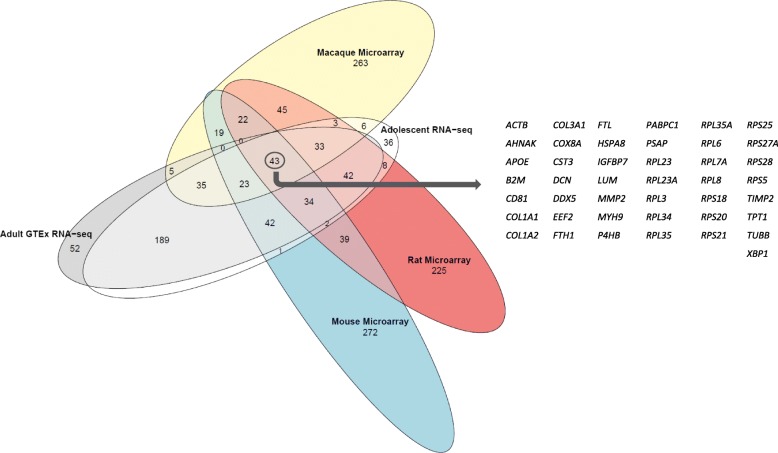
Venn diagram showing the 500 most highly expressed genes in mammary tissue from the 62 ADOL (“adolescent”) subjects in the current studies, 52 adult women (from GTEx), and from post-pubertal/young adult mice, rats, and macaques. For each group, genes with the highest median expression were selected after excluding mitochondrial genes. Mouse data is from whole inguinal mammary glands from 6-week-old female C57Bl/6 mice (*n* = 4), rat data is from whole glands from female peri-pubertal (postnatal day 42, *n* = 5) or late pubertal (postnatal day 63, *n* = 5) Sprague-Dawley rats, and macaque data is from female cynomolgus macaques (*n* = 4) who underwent breast biopsies 7–12-months after menarche (see also Additional File 1: Table S5)

We were motivated to characterize the transcriptome of the normal breast during adolescence and young adulthood because there is no existing data on this demographic. As the mouse is the most well-accepted animal model for studying human mammary gland development [3], we began by investigating the similarity of gene expression profiles across species using our human ADOL data and publicly available transcriptome data from mouse inguinal mammary glands collected at age 6 weeks [30], a developmental stage comparable to our post-menarchal ADOL subjects (Additional File 1: Table S5). Comparison of the 500 most highly expressed genes in breast tissue from ADOL and in post-pubertal/young adult mice [30] revealed that < 30% of genes overlapped. We expanded this comparative analysis to other existing genomic datasets: (1) RNA-seq data in GTEx from breast tissue samples in 52 adult women and (2) microarray data in GEO from mammary tissue in control or vehicle-treated post-pubertal macaques (7–12 months post-menarche) [31] and young adult rats (age 6–9 weeks) [32] (Additional File 1: Table S5). The adult women were 39.2 ± 8.6 years old (range 21–49), the majority (84.6%) were non-Hispanic Caucasian, and 34.6% were OB (mean BMI for cohort 26.4 ± 4.1 kg/m^2^, range 18.5–35.0), and the majority reported tobacco smoking and/or drinking. As shown in Fig. 2, the ADOL gene expression profile mapped most closely to that of adult women (88% overlap), whereas there was limited overlap (29–33%) between the ADOL dataset and data from other mammalian species. A set of 43 genes was shared by all groups (Fig. 2, Additional File 1: Table S6) and was enriched for two functional categories in DAVID: ribosomal proteins and extracellular matrix components. *Insulin-like growth factor-binding protein 7* (*IGFBP7*) was the only gene encoding a growth factor-related protein in this gene set, and there were no genes specific to sex steroid synthesis or signaling. Note that the results of these analyses were unchanged when we restricted the ADOL dataset to NOB participants.

### Effect of obesity on the whole transcriptome of the human breast

To determine whether body type might explain the variation in the ADOL dataset, we used principal component analysis (PCA) to visualize the variance in OB and NOB patient samples. The samples did not cluster by BMI category (Additional File 2: Fig. S1a), nor by any other available metadata (race, age, or tissue source). GTEx RNA-seq data from adult female breast tissue (*n* = 52) did not overlap with the ADOL data but showed a similar distribution along PC1 and PC2 (Additional File 2: Fig. S1b); after batch correction (i.e., sample source), however, the pattern of the ADOL samples and the GTEx adult breast samples became nearly indistinguishable (Additional File 2: Fig. S1c). We noted that, according to the histopathological data accompanying each GTEx specimen, the adult breast tissue samples showed considerable heterogeneity in cellular composition (e.g., “90% fibrous stroma” vs. “> 90% fat”), and we suspected that the percentage of adipose tissue might explain the observed variation in both our ADOL dataset and the adult breast GTEx data. Indeed, when we plotted the GTEx data from adult female subcutaneous adipose tissue (*n* = 35) alongside the ADOL and adult breast datasets, a subset of both ADOL and adult breast samples clustered with the subcutaneous adipose tissue (Additional File 2: Fig S1bc). As a negative control, we also plotted a GTEx stomach dataset but saw no clustering with the breast samples (data not shown).

To control for differences in breast tissue composition in NOB vs. OB subjects, we used CIBERSORT [23] to estimate the proportion of adipocytes in each sample. CIBERSORT is a computational method for estimating the fraction of cell subsets in bulk RNA-seq data from complex tissues, such as the breast, and has been validated using flow cytometry [23]. To virtually re-construct the cellular architecture of the breast, we input reference gene expression signatures for adipose tissue, breast epithelium, and breast fibroblasts (Additional File 1: Table S3). The adipose signature matrix was a modified version of the data generated by Glastonbury et al. [33] and includes CD4+ T cells [34], macrophages [35], human microvascular endothelial cells [36], and subcutaneous, mature adipocytes collected from the abdominal region in women undergoing elective abdominal surgery [37]. The CIBERSORT-estimated proportion of adipocytes in our ADOL samples (avg 0.3, range 0.008–0.8) correlated strongly with the position along PC1 (Additional File 2: Fig. S1d), and ADOL and adult breast GTEx samples with a higher estimated proportion of adipocytes were the closest to the cluster of subcutaneous adipose tissue samples (Additional File 2: Fig S1ef). Repeat analyses using different adipose tissue datasets [38, 39] produced similar results (data not shown).

We next performed differential gene expression analysis using DESeq2, comparing samples from NOB and OB ADOL participants while including the estimated proportions of adipocytes as a covariate in the design matrix. We used Ingenuity Pathway Analysis (IPA) to determine whether the observed differences in gene expression might represent a signature of activation or inhibition of one or more upstream transcriptional regulators in the OB tissue samples. IPA calculates whether a given set of DEGs and their direction of change are consistent with what is known about the activity of a particular transcriptional regulator and assigns two statistical measures, an overlap *p* value and *Z*-score. The *p* value indicates the degree of overlap between the DEG list and known targets of the regulator, whereas the *Z*-score indicates whether the direction of the regulator’s predicted effect (e.g., activates a target) matches the change in the expression of the target gene (e.g., target is upregulated). We first considered only upstream regulators meeting fairly stringent cutoffs (absolute Z-scores > 2 and *p* values < 0.05) and then mined the data for additional regulators within the pathways of interest.

Consistent with the typical clinical and biochemical phenotype of OB patients, our dataset of DEGs in breast tissue predicted inhibition of adiponectin (*Z*-score − 1.3) and rosiglitazone (*Z*-score − 0.3) action in OB compared with NOB ADOL samples Table 1, Additional File 1: Table S8).

**Table 1:** Upstream regulators identified by IPA having absolute *Z*-scores > 1 and predicted to be activated or inhibited in OB compared with NOB ADOL tissue samples

Activated		Inhibited
**Lipopolysaccharide**	Forskolin	LY294002
MAFB	**AKT1**	mir-21
**IL10**	**ESR1**	**ADIPOQ**
**CSF1**	**IL6**	GATA6
**IL10RA**	**CSF2**	IKZF1
TGM2	**TGFB1**	Immunoglobulin (Ig)
Progesterone	Tretinoin	
Dexamethasone	SPIB	
USP22	**poly rI:rC-RNA**	
**TCF4**	**HGF**	
**CCR2**	Tgf beta	
**Genistein**	**PGR**	
**Tetradecanoylphorbol acetate**	**ERBB3**	
**IL4**		

Regulators specifically mentioned in the text are in bold

**Fig. 3:**
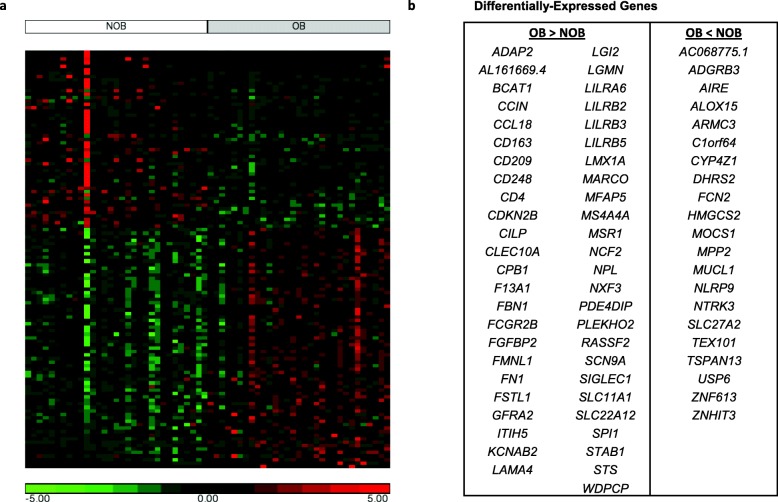
**a** Heatmap of differentially expressed genes in breast tissue from 31 overweight/obese (OB) compared with 31 non-overweight/obese (NOB) ADOL subjects. Columns are individual samples and rows are genes. Samples are grouped by body type (NOB or OB), and within each subgroup, samples are arranged left to right from those with the lowest to highest estimated adipocyte fraction. Genes are ranked by log_2_ fold change (highest at the bottom). Gene color and intensity represent normalized read counts corrected for the estimated proportion of adipocytes, log_2−_transformed, and standardized such that the mean of each row is 0 and the standard deviation is 1. **b** List of differentially expressed protein-coding genes in alphabetical order

In adult women, particularly postmenopausal women, obesity is believed to increase breast cancer risk through multiple mechanisms, including increased inflammation, growth factor secretion (e.g., FGF1), and local estrogen synthesis as well as an imbalance of adipokines and diminished PPAR-γ activity in the breast microenvironment [15, 40]. Our differential gene expression and IPA analyses therefore focused on these biological pathways. Using an FDR-adjusted *p* value < 0.05, we identified 69 upregulated and 51 downregulated genes (~ 60% protein coding) in samples from OB compared with NOB subjects (Additional File 1: Table S7, Fig. 3). IPA identified the cytokines CSF1 and IL-10 (and its receptor IL10R), the chemokine receptor CCR2, lipopolysaccharide, and TCF4 (also known as immunoglobulin TF2 or E2-2), a transcription factor which promotes expression of immunoglobulins [41] as among the most highly activated upstream regulators, suggesting a signature of inflammation in the OB breast tissue samples (Table 1, Additional File 1: Table S8). Tetradecanoylphorbol-acetate (TPA), an agent often used to induce cutaneous inflammation in animal models [42]; polyinosinic-polycytidylic acid [poly (rI:rC-RNA)], a synthetic dsRNA analogue and potent inducer of type 1 interferon [43]; and cytokines IL-4, IL-6, IL-13, CSF2, IFNG, and TGFβ-1 were also predicted to be upstream regulators with positive activation *Z*-scores (Table 1, Additional File 1: Table S8). CIBERSORT estimated that OB and NOB samples had comparable fractions of macrophages and T cells (0.6% vs. 0.4% and 3.1% vs. 2.8%, respectively), whereas the xCell average immune score was higher in OB compared with NOB (0.02 vs. 0.007, *p* = 0.02).

**Fig. 4:**
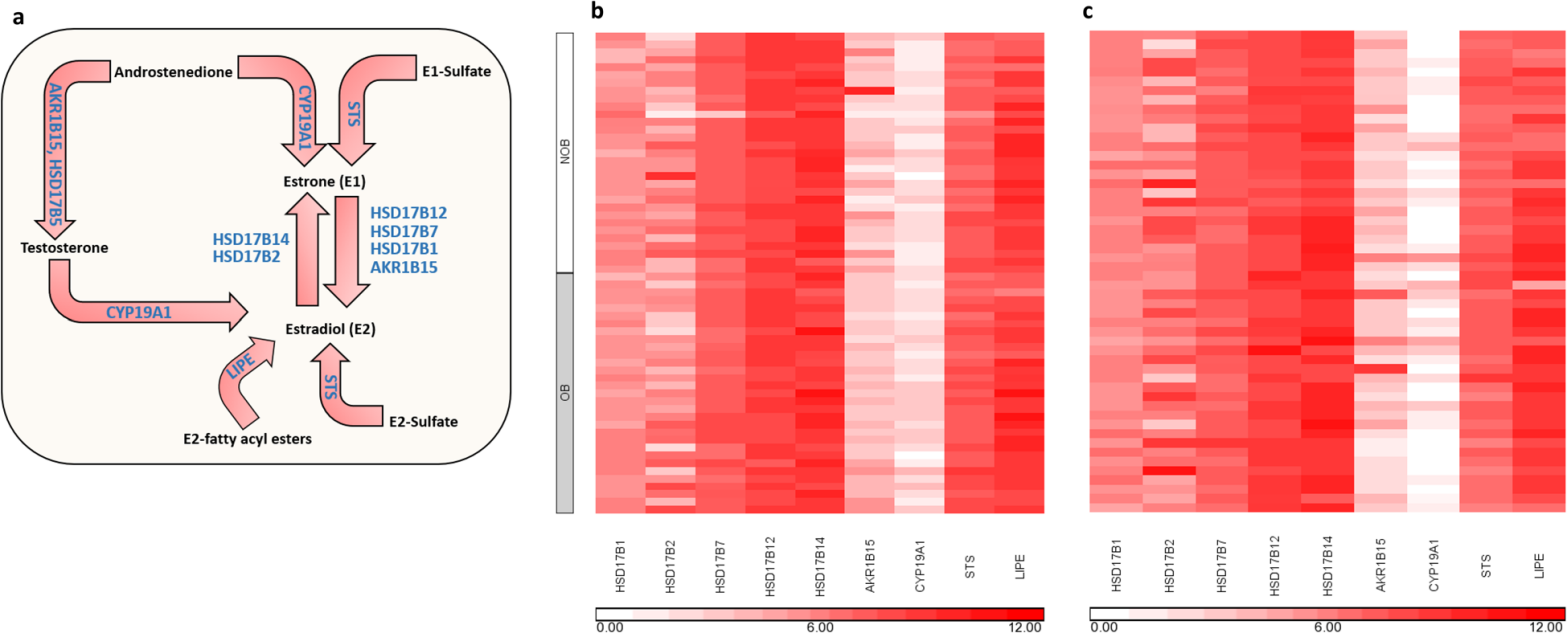
Abundance of genes within the estrogen synthesis pathway **a** in breast tissue samples from **b** 62 ADOL and **c** 52 adult women (data downloaded from GTEx). Rows are individual samples, and columns are genes. Color intensity represents the normalized read counts corrected for the estimated proportion of adipocytes, scaled per kb of transcript length, and log_2_-transformed. ADOL samples are grouped by body type (non-overweight/obese (NOB), overweight/obese (OB); see *y*-axis). For each ADOL subgroup and for adults, samples are arranged vertically from those with the highest to lowest estimated adipocyte fraction. HSD17B, hydroxysteroid 17-beta dehydrogenase; AKR1B15, Aldo-Keto reductase family member B15; CYP19A1, aromatase; STS, steroid sulfatase; LIPE, hormone-sensitive lipase

In obese women, inflammatory factors are believed to alter the steroidogenic enzyme profile of the breast, leading to increased local estrogen synthesis which has tumorigenic effects. For example, in breast cancer tissue: (1) the “sulfatase pathway” and “aromatase pathway” are upregulated, thereby providing a steady stream of precursors for estrogen synthesis in the form of inactive steroid sulfates or androgens, respectively, and (2) the reductive 17-β-hydroxysteroid dehydrogenases (17β-HSDs), which convert estrone (E1) into the more potent estrogen, estradiol (E2), are favored over the oxidative 17β-HSDs which inactivate E2 (reviewed in [44]) (Fig. 4a). In the current studies, we found no differences in steroidogenic enzyme mRNA expression in NOB and OB ADOL breast tissue samples with the exception of steroid sulfatase (*STS*), which was higher in OB samples (1.3-fold increase, *p*_adj_ = 0.02) (Fig. 4b). Aromatase (*CYP19A1*) expression was uniformly low, consistent with previous studies of normal adult breast tissue [45], and there appeared to be balanced expression of enzymes with oxidative (*HSD17B2*, *HSD17B14*) compared with reductive (*AKR1B15*, *HSD17B1*, *HSD17B7*, *HSD17B12*) activity. Hormone-sensitive lipase (*LIPE*) was highly expressed, with no difference between body weight groups. The relative abundance of genes involved in estrogen synthesis in the breast in ADOL was comparable to that of adult women (Fig. 4b, c).

While we did not observe overexpression of estrogen synthetic enzymes or classical estrogen receptor (ER) targets (e.g., *PR*, *AREG*, *GREB1*, or *TFF1*) (Additional File 1: Table S7) in breast samples from OB ADOL, several additional pieces of data suggested that signaling pathways downstream of the ER may, in fact, be activated in obesity. First, estrogen-bound ER-α represses its own gene (*ESR1*) expression [46], and *ESR1* expression tended to be lower (1.5-fold) in OB compared with NOB tissue samples (*p*_adj_ = 0.08). Second, IPA identified ER-α, genistein, and PR as activated upstream regulators (Table 1, Additional File 1: Table S8). Growth factors (VEGF, HGF), a growth factor receptor (HER3), and a kinase (AKT1) that are involved in hormone-independent activation of the ER (reviewed in [47]) were also among the upstream regulators with positive activation *Z*-scores (Table 1, Additional File 1: Table S8).

### Validating the effect of obesity on gene expression in the human breast using NanoString

We previously performed a pilot study in 30 of the same ADOL breast tissue samples (13 NOB, 17 OB) using a NanoString custom gene expression panel of 69 genes relevant to normal breast or breast cancer biology (Additional File 1: Table S2). Of note, > 60% of the genes in this code set were among the 43 genes that we also found to be highly expressed in breast tissue from ADOL, adult women, and other mammalian species. Of the genes with an absolute fold change in expression > 1.2 (in OB vs. NOB) in both the RNA-seq and NanoString datasets, 77% (23 of 30) changed in the same direction. Two genes in the custom panel were among the 120 genes determined to be differentially expressed in the RNA-seq analyses—*ESR1* (1.5-fold decrease, *p*_adj_ = 0.08) and *STS* (1.3-fold increase, *p*_adj_ = 0.02) (Additional File 1: Table S7)—and these genes showed a similar magnitude and direction of change in the NanoString analyses (*ESR1* 1.3-fold decrease, *STS* 1.2-fold increase). A comparison of fold changes for the full panel of 69 genes in OB relative to NOB samples as determined by RNAseq and NanoString is shown in Additional File 2: Fig. S2.

### Absence of immunohistochemical (IHC) signs of inflammation in adolescent breast tissue

**Fig. 5:**
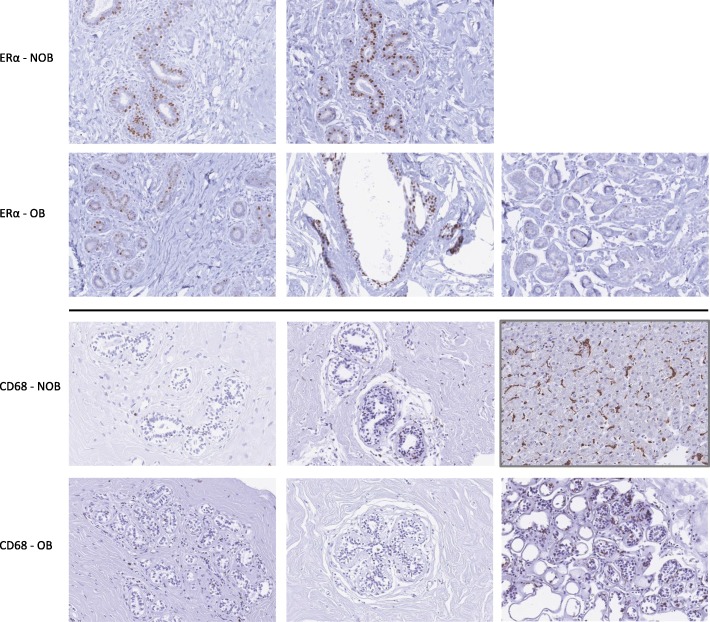
Representative pictures (× 20) of breast tissue from 2 non-overweight/obese (NOB) and 3 overweight (OB) ADOL subjects. Top: ER-α is expressed in a subset of ductal mammary epithelial cells. Bottom: no evidence of CD68+ macrophage infiltration in breast tissue samples. Note occasional non-specific staining of mast cells. Kupffer cells in human liver tissue (box outlined in gray) are shown as a positive control for CD68 staining

Crown-like structures (CLS), consisting of activated macrophages encircling dead or dying adipocytes, are a histopathologic sign of inflammation [48]. CLS have been identified in breast tissue of OB adult women with [49, 50] or without [51] breast cancer and also in benign breast tissue from adult women of normal weight but with signs of systemic metabolic dysfunction (e.g., insulin resistance, hypertriglyceridemia) [52]. We therefore investigated whether CLS might also be present in ADOL breast tissue. In a subset of samples in which paired paraffin-embedded tissue was available (*n* = 5; 3 OB, 2 NOB), we examined CD68-stained sections but did not identify any CLS (Fig. 5). We also performed IHC staining for ER-α and observed strong expression in a subset of epithelial cells lining the breast ducts (Fig. 5), consistent with previous studies in adult breast tissue [53]. We did not have a sufficient sample size to compare ER-α protein expression in OB and NOB subjects.

## Discussion

The current studies represent the first investigation of the immature human breast transcriptome during late puberty and early adulthood. The ADOL dataset revealed that, in the immature breast, at least three major pathways may be influenced by body weight—adipocyte function, inflammation, and ER activation. In the mid- to late reproductive years (30s–40s), the breast continues to undergo major cellular and architectural changes that are heavily influenced by menstrual history, parity, and lactation [54]. However, the current studies demonstrate that the transcriptome of the immature ADOL breast is actually more similar to the mature breast of adult women than to the immature breast of other mammalian species. This suggests that our understanding of human breast biology and pathology, which comes primarily from studies of adult female breast tissue, may be more relevant to the pubertal/early adult life stage than previously appreciated.

The pubertal/early adult life stage is particularly important because it represents one of the critical “windows of susceptibility” to breast cancer. During puberty, under the influence of estrogen, MECs proliferate at an exponential rate yet remain incompletely differentiated; the result is an accumulation of cells that are highly vulnerable to exposures from both the external environment and the microenvironment of the breast itself [5]. Secular changes in both the timing of pubertal milestones and the prevalence of pediatric obesity, two factors which influence future breast cancer risk in women, have made research into the biology of the ADOL breast all the more compelling.

Previous studies in OB children have identified inflammatory changes in subcutaneous [55] and visceral [56] adipose tissue and in the liver [57]. However, the reported association between pediatric obesity and decreased breast cancer risk in adulthood [16] raised the possibility that the breast may somehow be protected from obesity-related inflammation during childhood. The current studies suggest that the adolescent breast may not be completely spared from inflammation. We observed a strong molecular signature of cytokine and chemokine activation in breast tissue samples from OB participants and a higher xCell estimated immune score in OB, although these were not associated with gross histological inflammatory changes in the limited number of available samples. Together, these data suggest that in ADOL, there may be early signs of inflammation that portend the phenotypic changes in the breast of adult women with OB.

In OB postmenopausal women, inflammatory factors activate the aromatase and sulfatase pathways in breast tissue, leading to increased local estrogen synthesis and creating a nidus for breast cancer. In tissue samples from OB ADOL, we also observed signs of increased ER action (at the transcriptional level, by IPA) in the setting of increased inflammation. We did not observe upregulation of genes considered to be standard markers of ER action in the breast, such as *PR*, *AREG*, *GREB1*, or *TFF1* in OB samples. A recent RNAseq study of ER+ MECs isolated from normal breast tissue of three different women and grown in 3D cultures found that only three genes—*PR*, *LDL receptor-related protein 2* (*LRP2*), and *insulin-like growth factor-binding protein 4* (*IGFBP4*)—increased in all samples at 6 and 24 h after exposure to estrogen [58]. The observation that under identical culture conditions, a uniform population of cells (ER+ MECs) demonstrated extremely variable responses to estrogen suggests that in the current studies, differences in both the cellular composition of breast tissue samples and genetic background may have limited our ability to detect a molecular signature of ER activation shared across samples.

It is of interest that while studies of breast tissue from otherwise healthy OB women [51], OB women with breast cancer [49, 50, 59], and OB adult mice [60] have consistently demonstrated increased breast inflammation, an association between inflammation and estrogen synthesis and activity has only been observed in some studies. In a study of breast tissue from adult women (average age ~ 36 years) undergoing reduction mammoplasty, Sun et al. observed increased cytokine signaling and macrophage infiltration by differential gene expression and pathway analyses; genomic studies were bolstered by IHC studies demonstrating a greater number of CD68+ macrophage inflammatory foci (CLS) in breast tissue from OB than normal weight women. In these adult women, however, obesity-related inflammation was associated with downregulation of the ER, PI3-K/Akt, and ERK/MAPK signaling pathways [51]. Mullooly et al. [50] also observed increased CLS in benign breast tissue collected from OB breast cancer patients, and CLS were associated with a higher ratio of estrogen to androgen precursors in blood and breast tissue, suggesting increased aromatase activity. Heng et al. observed an enrichment of genes related to inflammation and to the early estrogen response pathway in a microarray study of ER+ breast tumor samples from OB compared with normal weight postmenopausal women enrolled in the Nurses’ Health Study, but this finding was not replicated in two other large breast cancer cohorts [59]. Lastly, in studies of mammary tissue from OB adult mice [60], pro-inflammatory factor abundance was also strongly correlated with aromatase activity and with expression of two classical ER target genes, *PR* and *TFF1*.

A number of studies have investigated the effects of a high-fat diet (HFD) on the mammary gland in mice when the diet is specifically initiated at puberty (~ 3 weeks of age), and these, too, have produced mixed results. In pubertal C57BL/6 mice, a HFD was associated with an accumulation of mammary CLS and diminished MEC proliferation and ductal elongation [61, 62]. Diet had no effect on mammary *ER* or *PRA* expression, but HFD-fed pubertal mice demonstrated a smaller increase in mammary *Areg* expression than control mice in response to a 5-day course of E2, suggesting that the observed defect in ductal growth was related to decreased estrogen responsiveness [62]. Similar dietary manipulation studies in pubertal BALB/c mice [63, 64] and FVB mice [61, 65], however, showed increased MEC proliferation with [61] or without [63] inflammation, and with no evidence for decreased estrogen responsiveness [62] among HFD-fed mice.

While it is well accepted that obesity-related inflammation promotes ER activity in the breast by increasing local E2 levels, obesity may also alter the profile of stromal growth factors that can activate the ER in the absence of its cognate ligand. Fibroblast growth factors, IGF-1, and EGF, for example, activate ER and/or its transcriptional co-activators through one or more kinase cascades (Ras/MAPK and PI3K/Akt) [66, 67]. Several of these growth factors and kinases were predicted to be activated in breast tissue samples from OB compared with NOB ADOL in the current study. As insulin can bind to the IGF1 receptor, it is possible that the hyperinsulinism associated with obesity also promotes hormone-independent ER activation in the breast. Taken together, the current studies in ADOL along with previous studies of the breast in obese women and mice suggest that the relationship between obesity and MEC proliferation, inflammation, and estrogen synthesis is complex and may relate to the timing and amount of weight gain, body fat distribution, changes in adipokines and growth factors, and genetic, nutritional, and other lifestyle factors.

We did not observe a unique transcriptomic signature of the young adult mammalian breast. The ADOL breast showed relatively little (< 30%) overlap with that of post-pubertal/young adult C57Bl/6 mice; however, this finding may not be generalizable to other mouse strains. The ADOL gene expression profile closely mirrored that of adult women. This was unexpected as the adult data was originally selected from the GTEx Portal based on female sex and age (20–49 years) alone. We later applied for and obtained additional phenotypic data on the female tissue donors through dbGaP (accession phs000424.v8.p2) and found that adult women were fortuitously of similar race and BMI to the ADOL. However, the majority of adult women endorsed smoking and drinking, two behaviors that not only increase breast cancer risk [68, 69] but are also less likely to be practiced by our younger (mostly underage) ADOL subjects. Further, we had no information on other relevant clinical characteristics such as the donor’s menopausal status, parity, and hormonal medication use.

In studies by Deroo et al. [70], physiological doses of estrogen were administered to ovariectomized prepubertal mice to mimic the natural course of mammary gland development (a 6-week process in mice). There was significant variability in gene expression profiles depending on the duration of estrogen exposure and mammary ductal length; the set of genes upregulated after 1 week of estrogen was distinct from those upregulated after 3 weeks of estrogen, and the gene sets mapped to unique functional pathways. Thus, it is quite possible that the human breast transcriptome is equally dynamic during development and that the expression profile of the breast in late puberty and young adulthood is very different from that of early puberty.

There was a small set of genes that was highly expressed in the breast of ADOL, adults, rodents, and macaques. This group of genes was enriched for ribosomal proteins, a finding that appears to be true for many unrelated human tissues (see GTEx top 50 for ovary, stomach, etc. at https://www.gtexportal.org/home/topExpressedGenePage), and for extracellular matrix (ECM) components. The ECM is known to play a critical role in breast morphogenesis, providing both mechanical and chemical cues that help to coordinate development of the diverse cell populations (e.g., MECs, stromal cells, adipocytes) of the breast microenvironment [71]. The overlap gene set included only one growth factor-related protein, IGFBP7. It is of interest that while *IGFBP7* is ubiquitously expressed in mice (ebi.ack.uk/gxa/genes), *Igfbp7*-null mice have a mammary-specific phenotype [72] (with the exception of an increased risk for liver and lung tumors [73]). *Igfbp7*^−/−^ mice are viable, fertile, and show no gross developmental anomalies, but they develop small mammary glands with fewer terminal end buds during puberty, malformed alveolar structures during pregnancy, and premature involution during lactation [72]. The role of IGFBP7 in human mammary development and disease may therefore deserve further attention.

While these studies represent the first investigation of the human ADOL breast at the molecular level, there are several important limitations. Given the difficulty in obtaining samples from this young demographic, nearly all of the samples were collected from ADOL undergoing breast reduction surgery. While this may limit generalizability, mammoplasty is a very common source of tissue among breast cancer researchers. We had no information on potentially relevant clinical characteristics such as patients’ menstrual cycle phase [74], hormonal medication use [74, 75], or gynecologic age (years since menarche); as these variables are not specifically related to body weight/fat, they are unlikely to have introduced systematic bias. Lastly, we attempted but ultimately failed to isolate MECs and adipocytes from breast tissue samples using laser capture microdissection. We therefore adjusted our bulk RNAseq analyses for differences in the cellular composition of breast tissue, as estimated by CIBERSORT, to avoid confounding. The CIBERSORT estimates were based on molecular signatures of most, but not all, cell types that comprise the human breast; we did not have data for mammary basal epithelial cells and the data for adipocytes was derived from subcutaneous abdominal adipose tissue rather than from breast adipose tissue. There has been a growing appreciation that in transcriptomic studies of complex human tissues, such as the blood [76], lung [77], and breast [78], cellular heterogeneity is an important source of expression variation, and if it is not accounted for, variation may be falsely attributed to the phenotypic variables under study (i.e., body weight). Studies in homogeneous cell populations isolated from adolescent breast tissue will be necessary to confirm our findings.

## Conclusions

We determined the molecular profile of the normal ADOL breast. Importantly, we found that in ADOL, as in adult women, obesity may be associated with increased inflammation and estrogen action within the breast microenvironment, at least at the transcriptional level. These data therefore suggest we reconsider the assertion, based on association studies, that pediatric obesity protects against breast cancer in adulthood.

## Supplementary information

**Additional file 1: Table S1.** Demographics associated with each sample, the source of the tissue (Boston Children’s Hospital, Baystate Medical Center, or the Cooperative Human Tissue Network), and the CIBERSORT-estimated fraction of adipocytes in each sample. Red indicates higher proportion of adipocytes and blue indicates lower proportion of adipocytes. Hisp = Hispanic, Cau = Caucasian, AA = African American. **Table S2.** Nanostring custom codeset panel of 69 genes. **Table S3.** Datasets utilized in the CIBERSORT deconvolution analysis. **Table S4.** Demographic characteristics and sample IDs for GTEx adult breast tissue and adult subcutaneous adipose tissue samples used in comparisons with ADOL RNA-seq data. **Table S5.** Mouse, rat, and macaque datasets used in cross-species comparisons and presented in Fig. 2. **Table S6.** List of genes (*n*=43) with high expression (in top 500) in breast tissue samples from ADOL, adult women, mouse, rat, and macaque. **Table S7.** Genes differentially expressed in OB compared with NOB ADOL breast tissue samples. **Table S8.** Upstream regulators identified by IPA with absolute Z-scores >1. Regulators mentioned in main text are in bold. Red - inflammatory, blue - estrogen signaling, purple - growth factors.

**Additional file 2: Figure S1.** Principal component (PC) analyses of ADOL dataset and adult GTEx RNA-seq data. (a) Initial analysis of breast tissue samples revealed no clear distinction by body type (NOB= non-overweight/obese, pink circles; OB=overweight/obese, green triangles). (b) Breast tissue samples in the ADOL dataset (*n*=62, pink circles) showed a similar dispersion along PC1 and PC2 as breast tissue samples from adult women in GTEx (*n*=52, blue squares), with a subset of samples clustering close to GTEx subcutaneous adipose tissue samples (*n*=35, green triangles). (c) The similarity between ADOL and adult breast tissue samples became more apparent after correction for a batch effect (sample source). (d) The CIBERSORT-based estimate of the proportion of adipocytes in each ADOL breast tissue sample explains sample distribution along the PC1 axis. Symbol shading represents the estimated fraction of adipose tissue from low (dark blue) to high (light blue). NOB = non-overweight/obese ADOL samples, OB = overweight/obese ADOL samples. (e) The estimated proportion of adipocytes in both ADOL (circles, current dataset) and adult breast tissue samples (squares, from GTEx) correlates with proximity to the cluster of subcutaneous adipose tissue samples on the far right (triangles, from GTEx). (f) Data shown in (e) after batch correction. **Figure S2.** Relative abundance of 69 genes assayed by both (A) RNA-seq (n=62 samples) and (B) NanoString (*n*=30 of the same 62 ADOL samples) technologies. Columns are individual samples and rows are genes. Samples are grouped by body type (NOB – non-overweight/obese, OB – overweight/obese; top), and within each subgroup, samples are arranged left to right from those with the lowest to highest estimated adipocyte fraction. Genes are ranked by log_2_fold-change (highest at bottom) as determined using RNA-seq data. For each gene, the color and intensity represent the abundance relative to the mean abundance for all 69 genes. Abundance was calculated using normalized read counts corrected for the estimated proportion of adipocytes, scaled per kb of transcript length, and log_2_ -transformed. (C) Correlation between log_2_fold changes in 69 genes as estimated by RNA-seq and NanoString in 30 matched ADOL samples. Values were corrected for the estimated proportion of adipocytes in each sample. (PPTX 224 kb)

## Data Availability

RNA-seq and NanoString data will be made available in a controlled-access NIH data repository.

## Abbreviations

ADOL: Adolescent and young adult subjects
BMI: Body mass index
CHTN: Collaborative Human Tissue Network
CLS: Crown-like structures
DEGs: Differentially expressed genes
ER-α: Estrogen receptor alpha
FDR: False discovery rate
GEO: Gene Expression Omnibus
GTEx: Genotype-Tissue Expression Portal
IPA: Ingenuity Pathway Analysis
MEC: Mammary epithelial cells
NOB: Non-overweight/obese
OB: Overweight/obese
PCA: Principal component analysis
