# Supplementary figures and images for: Investigation of the adolescent female breast transcriptome and the impact of obesity
Source: Breast Cancer Res. 2020 May 11;22:44. doi: 10.1186/s13058-020-01279-6 (PMC7216667; doi:10.1186/s13058-020-01279-6)

## Slide 1
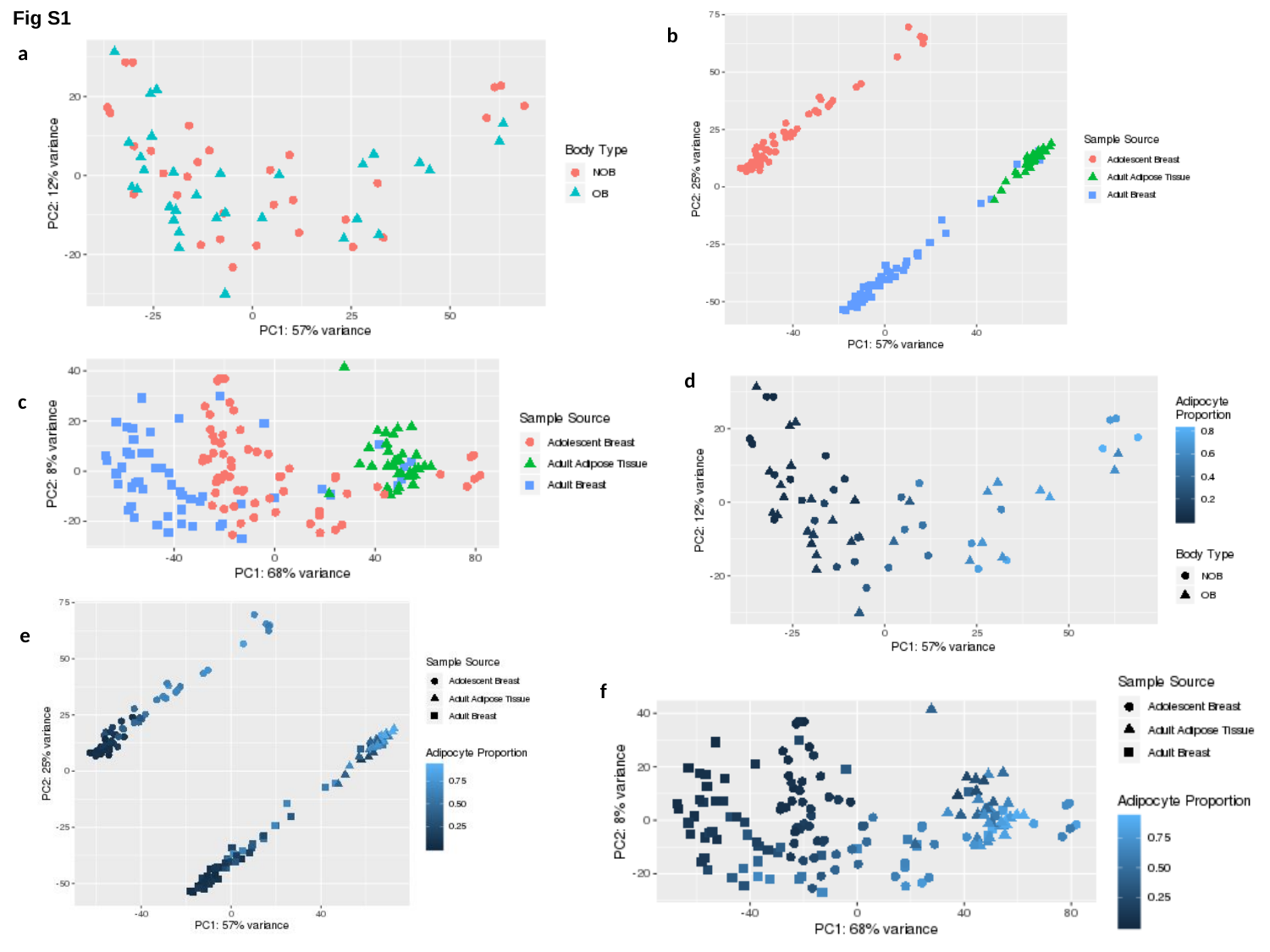

Fig S1
b
a
d
c
e
f

## Slide 2
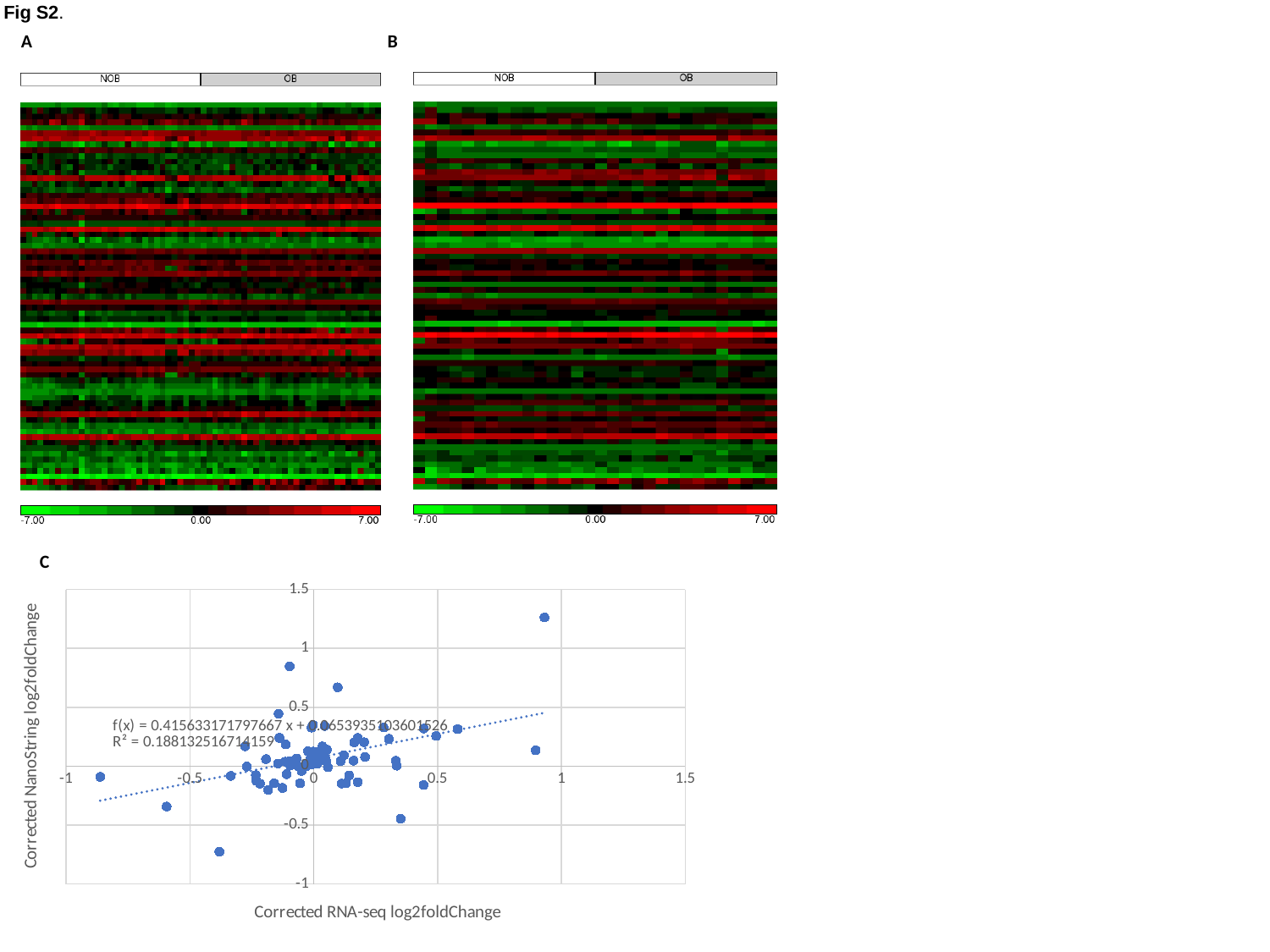

Fig S2.
A
B
C
### Chart
| Category | |
|---|---|

Supplement: Supplementary file 2 — Additional file 2: Figure S1. Principal component (PC) analyses of ADOL dataset and adult GTEx RNA-seq data. (a) Initial analysis of breast tissue samples revealed no clear distinction by body type (NOB= non-overweight/obese, pink circles; OB=overweight/obese, green triangles). (b) Breast tissue samples in the ADOL dataset (n=62, pink circles) showed a similar dispersion along PC1 and PC2 as breast tissue samples from adult women in GTEx (n=52, blue squares), with a subset of samples clustering close to GTEx subcutaneous adipose tissue samples (n=35, green triangles). (c) The similarity between ADOL and adult breast tissue samples became more apparent after correction for a batch effect (sample source). (d) The CIBERSORT-based estimate of the proportion of adipocytes in each ADOL breast tissue sample explains sample distribution along the PC1 axis. Symbol shading represents the estimated fraction of adipose tissue from low (dark blue) to high (light blue). NOB = non-overweight/obese ADOL samples, OB = overweight/obese ADOL samples. (e) The estimated proportion of adipocytes in both ADOL (circles, current dataset) and adult breast tissue samples (squares, from GTEx) correlates with proximity to the cluster of subcutaneous adipose tissue samples on the far right (triangles, from GTEx). (f) Data shown in (e) after batch correction. Figure S2. Relative abundance of 69 genes assayed by both (A) RNA-seq (n=62 samples) and (B) NanoString (n=30 of the same 62 ADOL samples) technologies. Columns are individual samples and rows are genes. Samples are grouped by body type (NOB – non-overweight/obese, OB – overweight/obese; top), and within each subgroup, samples are arranged left to right from those with the lowest to highest estimated adipocyte fraction. Genes are ranked by log2fold-change (highest at bottom) as determined using RNA-seq data. For each gene, the color and intensity represent the abundance relative to the mean abundance for all 69 genes. Abundance was calculate [file 13058_2020_1279_MOESM2_ESM.pptx]
